# Par-4/NF-*κ*B Mediates the Apoptosis of Islet *β* Cells Induced by Glucolipotoxicity

**DOI:** 10.1155/2016/4692478

**Published:** 2016-05-31

**Authors:** Wu QiNan, Gan XiaGuang, Lei XiaoTian, Deng WuQuan, Zhang Ling, Chen Bing

**Affiliations:** ^1^Endocrine Department, The First Affiliated Hospital of the Third Military Medical University, Chong Qing 400038, China; ^2^Outpatient Department, The First Affiliated Hospital of the Third Military Medical University, Chong Qing 400038, China

## Abstract

Apoptosis of islet *β* cells is a primary pathogenic feature of type 2 diabetes, and ER stress and mitochondrial dysfunction play important roles in this process. Previous research has shown that prostate apoptosis response-4 (Par-4)/NF-*κ*B induces cancer cell apoptosis through endoplasmic reticulum (ER) stress and mitochondrial dysfunction. However, the mechanism by which Par-4/NF-*κ*B induces islet *β* cell apoptosis remains unknown. We used a high glucose/palmitate intervention to mimic type 2 diabetes in vitro. We demonstrated that the high glucose/palmitate intervention induced the expression and secretion of Par-4. It also causes increased expression and activation of NF-*κ*B, which induced NIT-1 cell apoptosis and dysfunction. Overexpression of Par-4 potentiates these effects, whereas downregulation of Par-4 attenuates them. Inhibition of NF-*κ*B inhibited the Par-4-induced apoptosis. Furthermore, these effects occurred through the ER stress cell membrane and mitochondrial pathway of apoptosis. Our findings reveal a novel role for Par-4/NF-*κ*B in islet *β* cell apoptosis and type 2 diabetes.

## 1. Introduction

The basic pathogenic process of type 2 diabetes consists of islet *β* cell loss and dysfunction. As a result, most diabetes patients have very little *β* cell proliferation. Apoptosis is the main cause of islet *β* cell loss and dysfunction [1]; the overload of glucose and fatty acid induces the gathering of reactive oxide species (ROS), oxide-stress, and the increasing of ER stress and mitochondrial dysfunction attenuates the islet *β* cell apoptosis and dysfunction and contributes to the development of diabetes, the so called glucolipotoxicity [2–4].

Chronic, severe metabolic disorders induce ER stress and mitochondrial dysfunction and initiate the apoptosis pathway, including the cell membrane and mitochondrial pathways. This may be the common mechanism for islet *β* cell apoptosis, which is induced by glucolipotoxicity [5–7]. However, it is unclear what promotes ER stress and mitochondrial dysfunction in type 2 diabetes.

Prostate apoptosis response-4 (Par-4) is a novel proapoptosis factor that was initially discovered in prostate cancer. It contains a leucine zipper domain at the C terminus, which can bind with chaperones such as WT1 and protein kinase C. The selectivity for apoptosis induction in cancer cells (SAC) domain is a central domain that contains two nuclear localization sequences (NLS), NLS1 and NLS2, and a PKA phosphorylation site [8, 9]. Recent studies revealed that Par-4 can be secreted via the promotion of excessive ER stress. It interacts with FAS/ FASL and GRP78 in the cell membrane to activate caspase-8 and activate the initial cell membrane apoptosis pathway in the plasma. Par-4 is then cleaved by caspase-3, and this active fragment can translocate to the nucleus and induce apoptosis. Par-4 also attenuates cell apoptosis through the mitochondrial apoptosis pathway and can induce and amplify ER stress through this vicious cycle [10–12]. This suggests that Par-4 plays an important role in apoptosis. However, this phenomenon has not been observed in islet *β* cells.

Therefore, we hypothesized that ER stress triggers Par-4 secretion, causing it to translocate into the nucleus through the cell membrane and mitochondrial apoptosis pathways, inducing apoptosis in islet *β* cells [13]. We have identified Par-4 as a novel regulator of apoptosis in islet *β* cells which regulates and interacts with NF-*κ*B under high glucose and palmitate conditions.

## 2. Results

**Figure 1:**
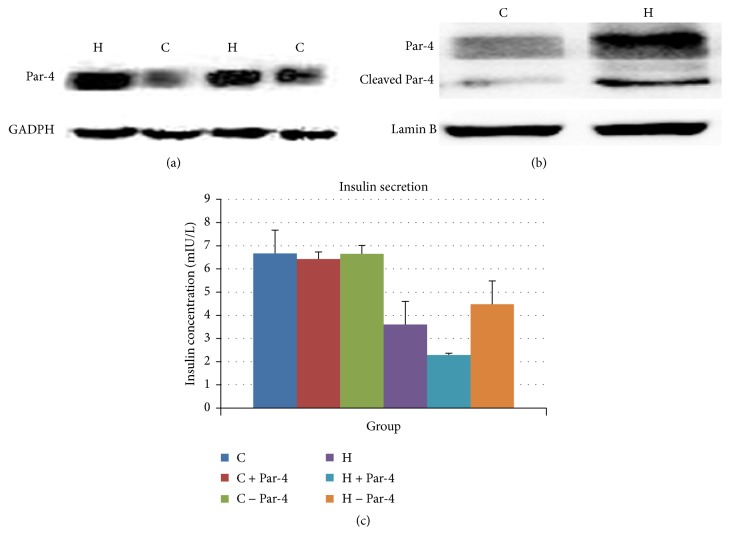
Par-4 is upregulated and secreted after glucose and palmitate cointervention in NIT-1.

We divided NIT-1 cells into six groups: a control group (Group C), high glucose/palmitate intervention group (Group H), overexpression in control group (Group C + Par-4), reduced expression Par-4 in control group (Group C − Par-4), overexpression of Par-4 in high glucose/palmitate intervention group (Group H + Par-4), and reduced expression Par-4 in high glucose/palmitate intervention group (Group H − Par-4). We analyzed Par-4 expression by western blot (WB) in NIT-1 cells and found that it was highly expressed after the high glucose/palmitate intervention compared with the control group (Figure 1(a)). Further analysis showed that Par-4 was abundantly expressed in the nuclei of NIT-1 cells after high glucose/palmitate intervention compared with the control group (Figure 1(b)). ELISA showed that Par-4 secretion is not affected by up- or downregulation of Par-4 under normal conditions, but Par-4 secretion is increased after high glucose/palmitate intervention compared with the control group. Overexpression of Par-4 after the high glucose/palmitate intervention can augment this process, and inhibition of Par-4 after the high glucose/palmitate intervention can attenuate this process (Figure 1(c)). These data suggested that the expression and secretion of Par-4 might be involved in the high glucose/palmitate intervention (Table 1).

**Figure 2:**
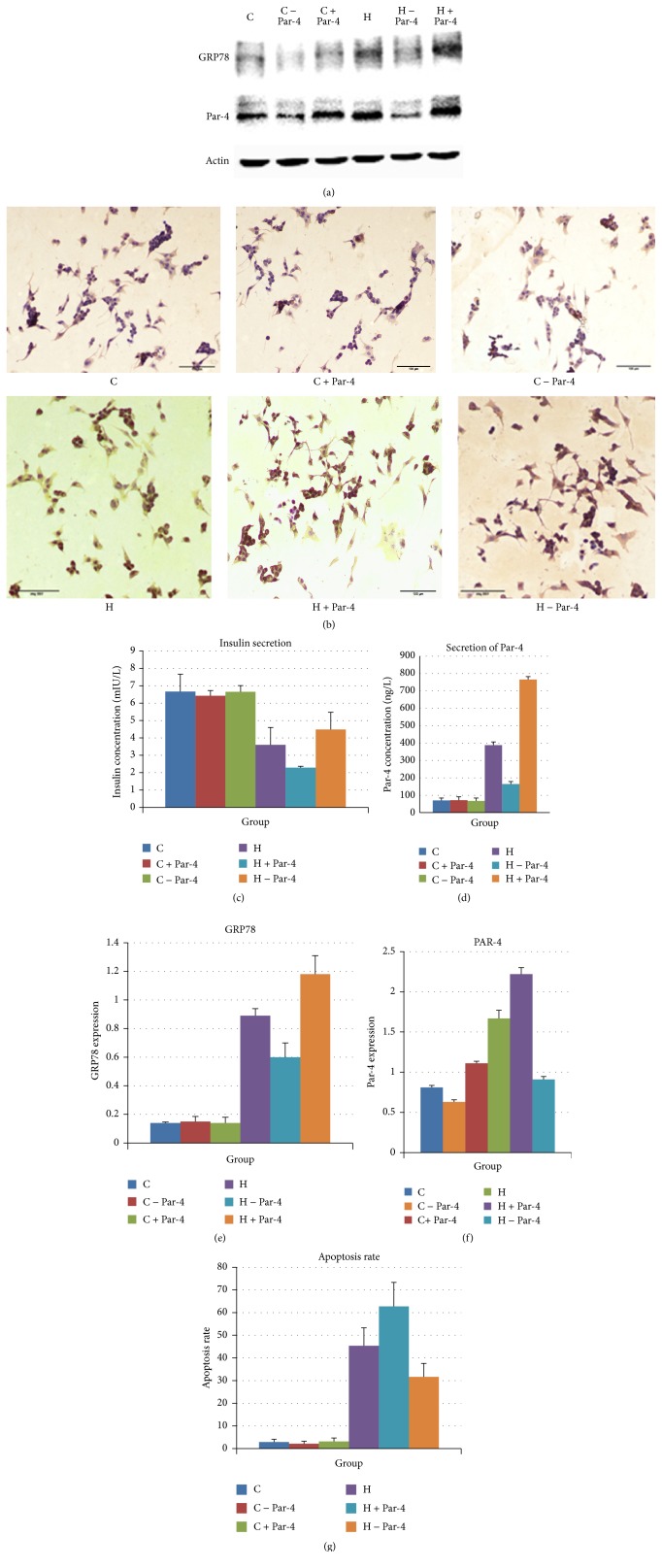
Par-4 is involved in the glucose and palmitate coinduced apoptosis of NIT-1 and insulin secretion.

WB showed that GRP78 is increased after the high glucose/palmitate intervention. Overexpression of Par-4 increased the expression of GRP78 (a parameter to evaluate ER stress levels) and reduced expression of Par-4 attenuated its expression in both the control group and the high glucose/palmitate intervention group (Figures 2(a), 2(e), and 2(f)). ELISA showed that the Par-4 concentration increased after the high glucose/palmitate intervention. Inhibition of Par-4 reduced Par-4 secretion in both the control group and the high glucose/palmitate intervention group (Figures 2(b) and 2(g)). After the high glucose/palmitate intervention, apoptosis was significantly elevated. Overexpression of Par-4 increased apoptosis and reduced expression of Par-4 attenuated apoptosis in the high glucose/palmitate intervention environment; however, neither overexpression nor inhibition of Par-4 had any effect on the apoptosis rate in the control group (Figures 2(c) and 2(d)). These data suggest that glucolipotoxicity increases ER stress, increases the secretion of Par-4, and induces apoptosis. Upregulation of Par-4 augments this process and inhibition of Par-4 attenuates this process. The results also indicate that glucolipotoxicity and ER stress play important roles in NIT-1 cell apoptosis and insulin secretion (Table 2).

**Figure 3:**
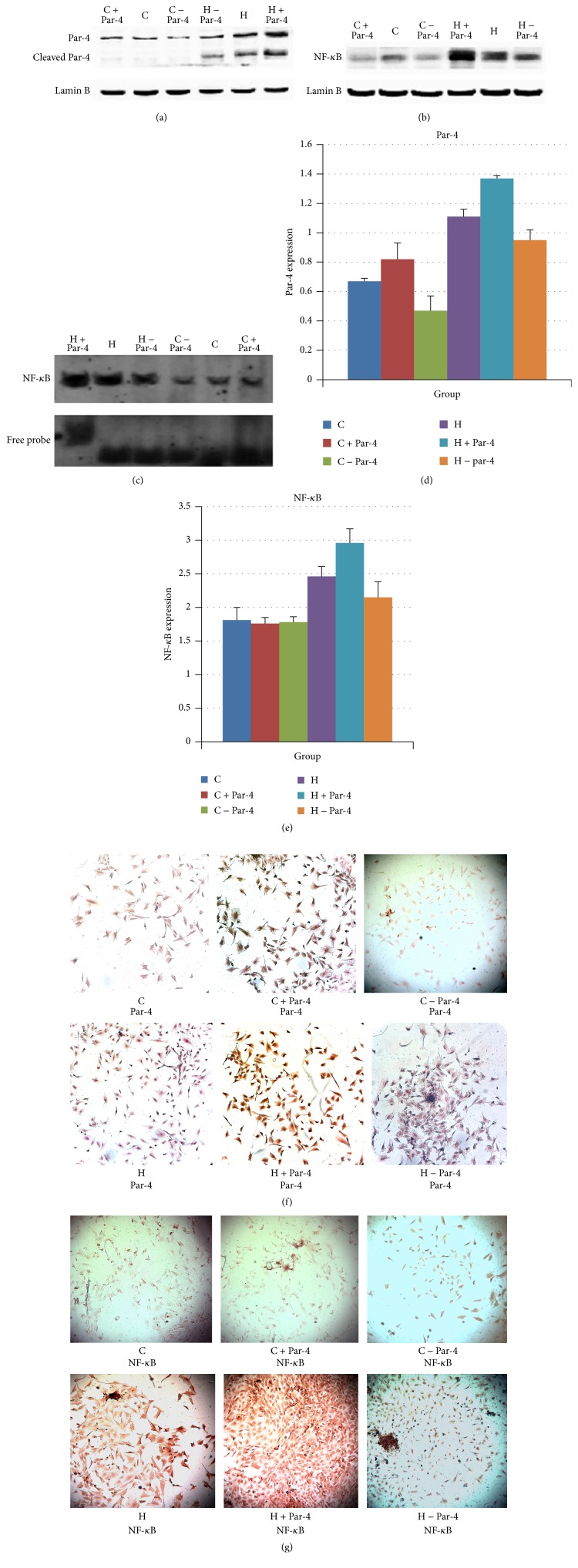
Glucose and palmitate cointervention activates Par-4/NF-*κ*B to induce apoptosis.

WB of nuclear proteins showed that Par-4 and NF-*κ*B expression were increased after the high glucose/palmitate intervention. Overexpression of Par-4 increased Par-4 and NF-*κ*B expression and downregulation of Par-4 attenuated Par-4 and NF-*κ*B expression in the high glucose/palmitate intervention group. However, neither overexpression nor inhibition of Par-4 had any effect on the expression of the Par-4, cleaved Par-4, or NF-*κ*B in the control group in the nucleus (Figures 3(a), 3(b), 3(d), and 3(e)). Immunohistochemistry showed that after the high glucose/palmitate intervention, expression of Par-4 and NF-*κ*B increased in the nucleus. Overexpression of Par-4 increased the expression of NF-*κ*B and downregulation of Par-4 decreased the expression of NF-*κ*B in the high glucose/palmitate intervention group (Figures 3(f) and 3(g)). EMSA indicated that the transcription level of NF-*κ*B was increased after the high glucose/palmitate intervention. Overexpression of Par-4 increased NF-*κ*B expression and downregulation of Par-4 attenuated NF-*κ*B expression in the high glucose/palmitate intervention group. However, neither overexpression nor inhibition of Par-4 had any effect on the transcription level of NF-*κ*B in the control group (Figure 3(c)). These data suggest that glucolipotoxicity increases Par-4 and NF-*κ*B expression in the nucleus and induces apoptosis. Upregulation of Par-4 expression can augment this process, whereas inhibition of Par-4 can attenuate this process. The results also indicate that Par-4 and NF-*κ*B play important roles in the nucleus in NIT-1 cell apoptosis (Table 3).

**Figure 4:**
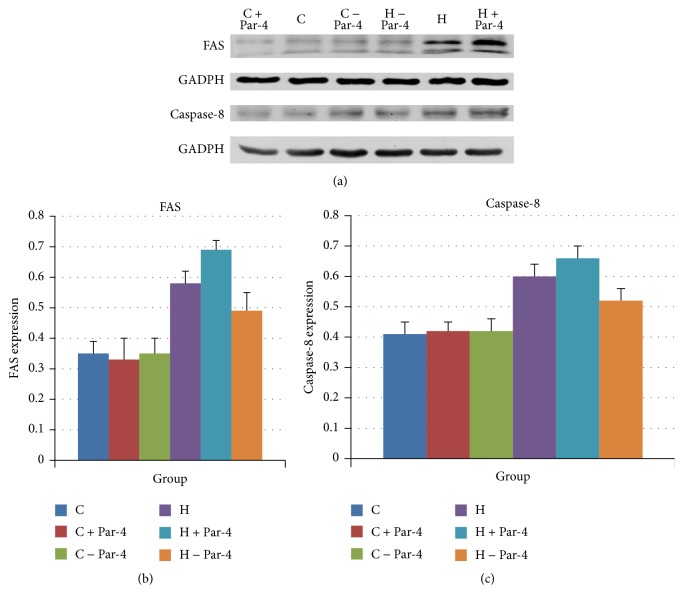
Glucose and palmitate cointervention activates Par-4 and mediates the cell membrane apoptotic pathway to induce NIT-1 apoptosis.

WB showed that FAS and caspase-8 were increased after the high glucose/palmitate intervention. Overexpression of Par-4 increased FAS expression and downregulation of Par-4 attenuated FAS expression in the high glucose/palmitate intervention group. However, neither overexpression nor downregulation of Par-4 had any effect on the expression of FAS in the control group (Figures 4(a) and 4(b)). Caspase-8 increased after the high glucose/palmitate intervention. Overexpression of Par-4 increased caspase-8 expression and downregulation of Par-4 attenuated caspase-8 expression in the high glucose/palmitate intervention group. However, neither overexpression nor inhibition of Par-4 had any effect on the expression of caspase-8 in the control group (Figures 4(a) and 4(c)). These data suggest that glucolipotoxicity increased the expression of FAS and caspase-8, which are the key factors in the cell membrane mediated apoptotic pathway. There is increased cleavage of Par-4 in the nucleus. Upregulation of the expression of Par-4 can augment this process and inhibition of Par-4 can attenuate this process (Table 4).

**Figure 5:**
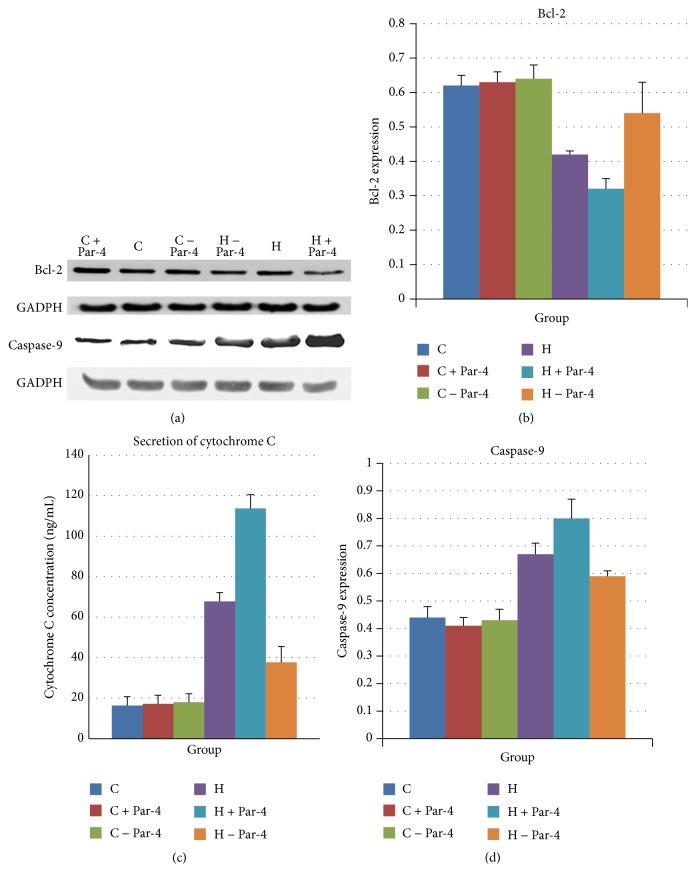
Glucose and palmitate cointervention activates Par-4 via the mitochondrial apoptotic pathway to induce NIT-1 apoptosis.

WB showed that caspase-9 was increased after the high glucose/palmitate intervention. Overexpression of Par-4 aggravated the expression of caspase-9 and downregulation of Par-4 attenuated caspase-9 expression in the high glucose/palmitate intervention group. However, neither overexpression nor inhibition of Par-4 had any effect on the expression of caspase-9 in the control group (Figures 5(a) and 5(b)). Bcl-2 expression was decreased after the high glucose/palmitate intervention. Overexpression of Par-4 augmented the decreased expression of bcl-2 and downregulation of Par-4 attenuated the decrease in bcl-2 expression in the high glucose/palmitate intervention group. However, neither overexpression nor inhibition of Par-4 had any effect on the expression of Bcl-2 in the control group (Figures 5(a) and 5(d)). ELISA showed that the concentration of cytochrome C increased after the high glucose/palmitate intervention. Downregulation of Par-4 decreased the concentration of cytochrome C. However, neither overexpression nor inhibition of Par-4 had any effect on the concentration of cytochrome C in the control group (Figure 5(c)). These data suggest that glucolipotoxicity increased caspase-9 and cytochrome C expression. These are the key factors of the mitochondrial apoptotic pathway. Furthermore, overexpression of Par-4 can augment this process, whereas downregulation of Par-4 can attenuate this process (Table 5).

PDTC inhibits NF-*κ*B expression and efficacy. We divided NIT-1 cells into five groups: the control group (Group C), the high glucose/palmitate intervention group (Group H), the high glucose/palmitate and PDTC intervention group (Group HP), the overexpression of Par-4 and high glucose/palmitate intervention group (Group H + Par-4), and the overexpression of Par-4 and high glucose/palmitate and the PDTC intervention group (Group HP + Par-4). We determined the rate of apoptosis in each group by TUNEL staining. We also determined the nuclear protein expression of NF-*κ*B and Par-4 by WB and the transcriptional activity of each group by EMSA.

**Figure 6:**
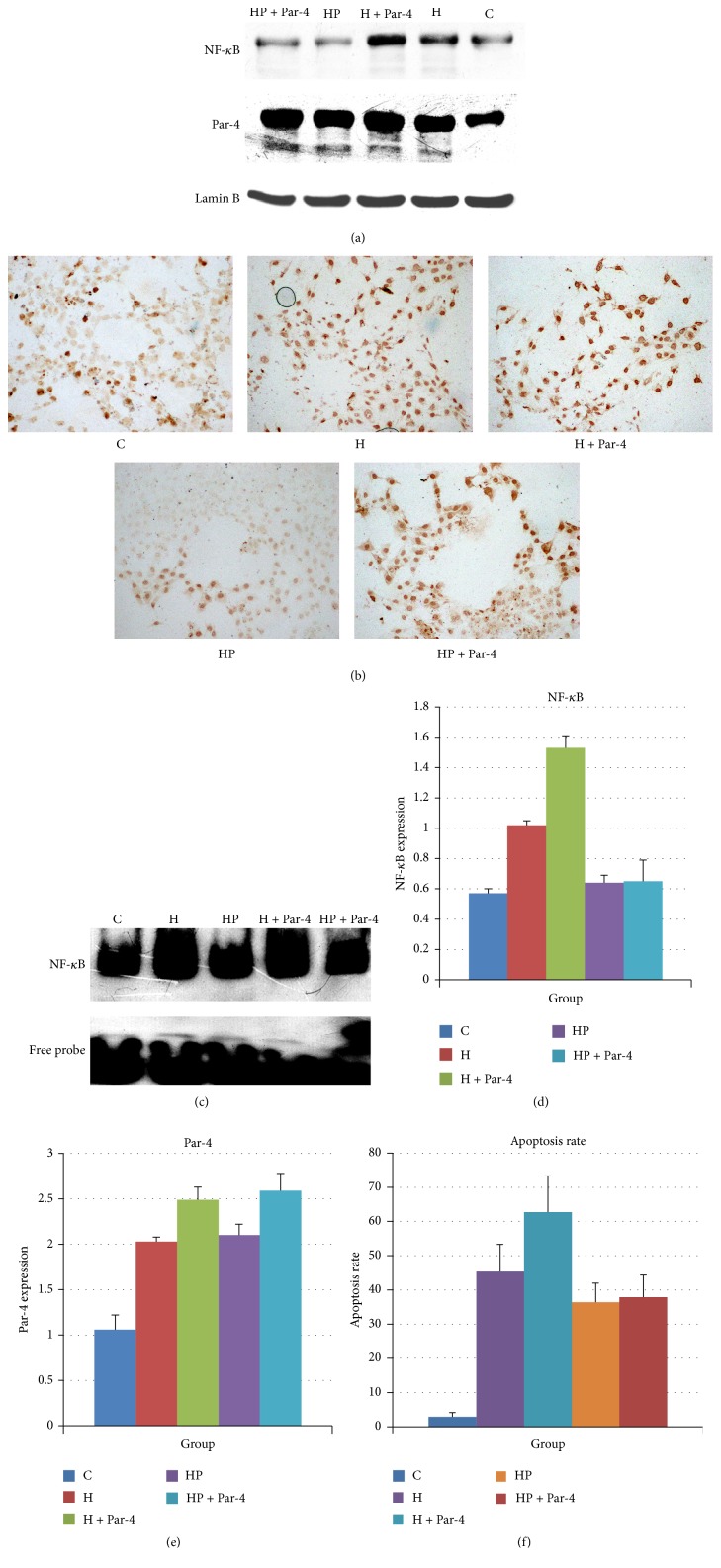
Inhibition of NF-*κ*B reduced apoptosis, which was induced by overexpression of Par-4 in high glucose/palmitate intervention NIT-1 cells.

Par-4 expression and the rate of apoptosis were increased in Group H and Group H + Par-4 compared with Group C. When Par-4 was upregulated, the rate of apoptosis increased in Group H + Par-4 compared with Group H (Figures 6(b) and 6(f)). After the PDTC intervention, there was no significant difference in the expression of Par-4 between Group H and Group HP. Group H + Par-4 and Group HP + Par-4 (Figures 6(a) and 6(e)) also exhibited no difference in Par-4 expression. The expression and transcriptional activity of NF-*κ*B were decreased after the PDTC intervention. The rate of apoptosis was also increased after this intervention (Figures 6(a), 6(b), and 6(c)). These data suggest that the induction of apoptosis by Par-4 may depend on the expression and transcriptional activity of NF-*κ*B (Table 6).

At first, the cultured NIT-1 cells were divided into six groups: the control group (Group C), the high glucose/palmitate intervention group (Group H), the overexpression in control group (Group C + Par-4), the downregulation Par-4 in control group (Group C − Par-4), the overexpression of Par-4 in high glucose/palmitate intervention group (Group H + Par-4), and the downregulation Par-4 in high glucose/palmitate intervention group (Group H − Par-4). We determined the rate of apoptosis of the cells by TUNEL staining and the nuclear protein expression by WB. Compared to the C group, the C + Par-4 and C − Par-4 groups demonstrated no significant differences in the rate of apoptosis (*P* > 0.05). This result indicates that, under normal physiological conditions, Par-4 overexpression or downregulation does not affect apoptosis. This phenomenon holds true in the knock-out and knock-in Par-4 mouse models in previous studies [11].

The rate of apoptosis in the H group was significantly higher than that of the C group (*P* < 0.05). Compared to the H group, the rate of apoptosis in the H + Par-4 group was significantly increased (*P* < 0.05), and the rate of apoptosis in the H − Par-4 group was significantly decreased (*P* < 0.05). These results suggested that the high glucose/palmitate intervention can induce NIT-1 cell apoptosis, and under conditions with high glucose or palmitate, overexpression of Par-4 can significantly increase the apoptosis rate, whereas downregulation of Par-4 can decrease apoptosis.

To determine the level of endoplasmic reticulum (ER) stress in each group, we determined the total cell protein expression of GRP78 in each group by WB. We also determined the Par-4 concentration in the supernatant for each group by ELISA. The results indicated that compared to the C group, GRP78 protein expression in the C + Par-4 and C − Par-4 groups exhibited no significant difference (*P* > 0.05). The Par-4 concentration in the supernatant also was not significantly different (*P* > 0.05). Previous studies suggested that endoplasmic reticulum stress induced by high glucose and fatty acid levels is the main cause of the islet cell apoptosis. Previous work has also shown that ER stress can increase the secretion of Par-4. However, in this study, overexpression and downregulation of Par-4 in normal cells did not change the level of ER stress under the normal environment. Therefore, Par-4 secretion and rate of apoptosis were unchanged. However, in the H, H + Par-4, and H − Par-4 groups, GRP78 protein expression was significantly higher than that in group C (*P* < 0.05). The rate of apoptosis and the Par-4 concentration in the supernatant were significantly increased (*P* < 0.05). Compared to group H, GRP78 protein expression was significantly increased in the H + Par-4 group (*P* < 0.05). The rate of apoptosis and the Par-4 concentration in the supernatant were significantly increased (*P* < 0.05). GRP78 protein expression was significantly decreased (*P* < 0.05) in the H − Par-4 group, and the rate of apoptosis and the Par-4 concentration were significantly decreased (*P* < 0.05). These results suggest that the high glucose/fatty acids intervention increases the level of ER stress, thereby increasing Par-4 secretion and inducing apoptosis. Overexpression of Par-4 can increase the levels of ER stress and secretion of Par-4, thereby increasing the rate of apoptosis. Downregulation of Par-4 can reduce the levels of endoplasmic reticulum stress and Par-4 secretion, thereby decreasing the rate of apoptosis in islet *β* cells.

NF-*κ*B is a common nuclear factor that regulates apoptosis and cell survival in many diseases. The high glucose/palmitate intervention increases the expression of NF-*κ*B and the rate of apoptosis in islet *β* cells. There may be some correlation between NF-*κ*B expression and islet *β* cell apoptosis, but the specific relationships and mechanism are unclear [14]. We know that Par-4 can inhibit NF-*κ*B, inducing apoptosis in many cancer cells. Does this phenomenon exist in islet *β* cells?

In our study, we detected nuclear protein NF-*κ*B expression and nuclear protein NF-*κ*B activity to demonstrate the relationship between NF-*κ*B and Par-4 during the apoptosis process in NIT-1 cells. Compared to the C group, no significant difference in the protein expression and activity of NF-*κ*B existed between the C + Par-4 and C − Par-4 groups (*P* > 0.05). These results indicate that, under normal conditions, overexpression and downregulation of Par-4 expression do not affect the protein expression and activity of NF-*κ*B in the nucleus or the rate of apoptosis. In the H, H + Par-4, and H − Par-4 groups, the protein expression and activity of NF-*κ*B were significantly higher than the C group (*P* < 0.05). Compared to the H group, overexpression of Par-4 significantly increased the protein expression and activity of NF-*κ*B in the H + Par-4 group. It also increased the apoptosis rate (*P* < 0.05). Inhibition of Par-4 significantly decreased the protein expression and activity of NF-*κ*B in the H − Par-4 group. It also decreased the apoptosis rate. These results indicate that Par-4 increases apoptosis and the nuclear expression and activity of NF-*κ*B, which is induced by high glucose and palmitate in NIT-1 cells. Downregulation of Par-4 can reduce the expression and activity of NF-*κ*B and decrease the rate of apoptosis induced by the high glucose/palmitate intervention in NIT-1 cells.

To investigate the relationship between Par-4 and NF-*κ*B in apoptosis in islet *β* cells, we further divided NIT-1 cells into five groups: the control group (Group C), the high glucose/palmitate intervention group (Group H), the high glucose/palmitate and PDTC intervention group (Group HP), the overexpression of Par-4 and high glucose/palmitate intervention group (Group H + Par-4), and the overexpression of Par-4 and high glucose/palmitate and also PDTC intervention group (Group HP + Par-4). We used TUNEL staining to detect the rate of apoptosis, WB to detect the protein expression of Par-4 and NF-*κ*B, and EMSA to detect the transcription activity level of NF-*κ*B. The results showed that, compared to Group C, the apoptosis rate and expression of Par-4 and NF-*κ*B were increased in Group H. Compared to Group H, overexpression of Par-4 increased the expression and activity of NF-*κ*B and the rate of apoptosis in the H + Par-4 group (*P* < 0.05). However, after inhibition of NF-*κ*B by PDTC, although there was no significant difference in Par-4 expression compared to the H group, the apoptosis rate and protein expression and activity of NF-*κ*B were significantly decreased in the HP group (*P* < 0.05). Similarly, compared to the H + Par-4 group, although there was no significant difference in Par-4 expression, the rate of apoptosis and the protein expression and activity of NF-*κ*B were decreased significantly in the HP + Par-4 group (*P* < 0.05). These results suggested that the mechanism by which Par-4 induces apoptosis involves directly inhibiting the expression and activity of NF-*κ*B in the nuclei of NIT-1 cells.

Previous studies have indicated that Par-4 induces apoptosis through extracellular and intracellular pathways. ER stress increases Par-4 secretion and activates the cell membrane apoptosis pathway, also called the extracellular pathway. The intracellular pathway operates via the upregulation of cytosolic Par-4, thereby blocking the expression of Bcl-2 and activating mitochondrial dysfunction to induce apoptosis. Through the previously described experiments, we revealed that the Par-4-induced apoptosis effect in NIT-1 cells is associated with ER stress, which is induced by the high glucose/palmitate intervention. Without endoplasmic reticulum stress, both upregulation and downregulation of Par-4 expression in the cytoplasm had no effect on the apoptosis of NIT-1 cells. This suggested that the Par-4-induced apoptosis in NIT-1 cells may occur through the extracellular pathway. We then investigated key markers of the cell membrane pathway, such as FAS and caspase-8, by WB. Compared to the C group, the H, H + Par-4, and H − Par-4 groups exhibited significantly increased expression of caspase-8 and FAS (*P* < 0.05). Compared to the H group, the protein expression of caspase-8 and FAS was significantly increased in the H + Par-4 group (*P* < 0.05). Compared to the H group, the protein expression of caspase-8 and FAS was significantly decreased in the H − Par-4 group (*P* < 0.05). Taken together, the above results indicate that the effects of Par-4-induced apoptosis are associated with ER stress and the cell membrane pathway of apoptosis in NIT-1 cells. Under the high glucose/palmitate intervention environment, overexpression of Par-4 can further promote apoptosis via this pathway and downregulation of Par-4 can reduce apoptosis.

Previous studies have indicated that the mitochondrial pathway is one of the ways in which apoptosis is induced through the activation of Par-4. We detected the expression of caspase-9 and Bcl-2, the key enzymes of the mitochondrial apoptosis pathway, by WB and detected the secretion of cytochrome C by ELISA. Compared to the C group, the protein expression of caspase-9 and secretion of cytochrome C were significantly higher, and the protein expression of Bcl-2 was significantly decreased in the H, H + Par-4, and H − Par-4 groups (*P* < 0.05). Compared to the H group, the protein expression of caspase-9 and secretion of cytochrome C were significantly increased, and the protein expression of Bcl-2 was significantly decreased in the H + Par-4 group (*P* < 0.05). Compared to the H group, the protein expression of caspase-9 and secretion of cytochrome C were significantly decreased, and the protein expression of Bcl-2 was significantly increased in the H + Par-4 and H − Par-4 groups (*P* < 0.05). These results indicate that Par-4 is activated under the high glucose/palmitate intervention, leading to mitochondrial dysfunction in NIT-1 cells, which induces apoptosis. Increased Par-4 expression can enhance the degree of mitochondrial dysfunction and promote the apoptosis of NIT-1 cells. Downregulation of Par-4 can significantly decrease the degree of mitochondrial dysfunction, thereby decreasing apoptosis in NIT-1 cells. The results above suggest that, under the high glucose/palmitate intervention, Par-4-induced islet *β* cell apoptosis may also involve the mitochondrial pathway.

## 3. Discussion

### 3.1. Apoptosis in Islet *β* Cells Plays an Important Role in the Pathogenesis of Type 2 Diabetes

According to the latest epidemiologic studies, there are more than one hundred million adult patients with diabetes in China. The prevalence of diabetes has risen to 11.6%, and more than 90% of these patients have type 2 diabetes [15]. Due to this high disease burden, prevention and treatment of diabetes are of the utmost importance. The UKPDS study found that patients with type 2 diabetes have only 50% of islet *β* cell function. This is one of the major reasons for poor glycemic control and is the major pathophysiological basis for the acute and chronic complications in type 2 diabetes [4, 5]. Animal models and human autopsies have revealed that the reason underlying islet *β* cell function decline in type 2 diabetes is significantly reduced *β* cell mass. Inflammatory necrosis, differentiation and dysfunction of *β* cells, abnormal apoptosis in *β* cells, and reduction in the amount of islet *β* cells are important factors in the pathogenesis of type 2 diabetes mellitus [1, 16, 17]. Treatment with insulin may improve hyperglycemia, but it cannot protect the body from chronic exposure to high glucose levels. This results in serious and irreversible disability and lethal complications. Although the specific mechanism of apoptosis in type 2 diabetes islet *β* cells is not clear, it is at least suggested that effective inhibition of the apoptosis of islet *β* cells is one of the key steps in the prevention of type 2 diabetes.

### 3.2. ER Stress and Mitochondrial Dysfunction Are the Main Mechanisms of *β* Cell Apoptosis in Type 2 Diabetes

Accumulating evidence has revealed that glucose and lipid metabolic disorders may be caused by exposure to a high glucose and high fatty acid environment. Amyloid deposition and inflammatory cytokines can lead to ER stress and mitochondrial dysfunction and initiate apoptosis signaling pathways, including the cell membrane and the mitochondrial pathways. Researchers believed that regulation of the apoptotic signaling pathways (including cell membrane and mitochondrial signaling pathways) plays an important role in the development of pancreatic *β* cells in diabetes [1, 18–21]. Therefore, the key to the prevention and cure of diabetes is reducing the activation of the apoptotic pathway in islet *β* cells.

### 3.3. Par-4 Can Induce Apoptosis in Tumor Cells through ER Stress

Prostate apoptosis response protein-4 (Par-4) is a proapoptotic factor located on human chromosome 12q21. This study found that the proapoptotic effects of Par-4 are not limited to tumor cells but are also involved in a variety of other processes [22, 23]. A variety of factors can activate the NLS of the N terminus such that Par-4 can be transferred to the nucleus and activated [8, 9]. Endogenous Par-4 activation induces apoptosis in tumor cells, but this does not occur in normal cells and immortalized cells. The effect also does not rely on the existence of a leucine zipper. Scholars have described this phenomenon as selectively induced apoptosis in tumor cells (SAC). In hormone-dependent cancer cells, the FAS/ FASL complex translocates to the cell membrane, and the death inducing signaling complex (DISC) forms, resulting in activation of the caspase cascade effect [11, 12]. In the end, the cleavage of caspase-3 in the nucleus causes Par-4 to downregulate the expression of NF-*κ*B and promotes apoptosis [10]. The main apoptosis pathways are the cell membrane pathway and mitochondrial apoptosis pathway. To induce apoptosis, Par-4 binds to GRP78 on the cell membrane to initiate ER stress. ER stress can also increase Par-4 secretion; therefore, it is a vicious cycle for the continuous induction of cell apoptosis. On the other hand, Par-4 can also be induced through the mitochondrial pathway after caspase-3 cleavage in the nucleus [10–12]. ER stress and mitochondrial dysfunction are commonly involved in the pathophysiological basis of many age-related diseases such cancer, Alzheimer's disease, and some cardiovascular diseases. Researchers found that Par-4 may participate in the pathophysiology of these diseases [13, 24]. This is the first report linking Par-4 to islet *β* cells.

### 3.4. Par-4/NF-*κ*B Induced Apoptosis of Islet *β* Cells under the High Glucose/Palmitate Intervention via the ER Stress Cell Membrane and Mitochondrial Pathways

Par-4 is widely expressed in various types of cells. A number of studies also suggest that it can participate in a variety of cell apoptosis processes via the cell membrane and mitochondrial pathways. Our study strongly suggested that it might be involved in islet *β* cell apoptosis.

1. According to previous studies, there is a close relationship between Par-4 and ER stress and ER stress may be one of the mechanisms of *β* cell apoptosis in type 2 diabetes. We therefore hypothesized that Par-4 may be involved in the pathogenesis of apoptosis of islet *β* cells via ER stress, which is induced by type 2 diabetes [13, 25].
2. Our experiments found that upregulation and downregulation of Par-4 in normal islet *β* cells do not induce apoptosis; however, under high glucose/palmitate intervention-induced endoplasmic reticulum stress, Par-4 expression levels were increased and induced NIT-1 cell apoptosis. The apoptosis rate and ER stress levels were all increased and positively correlated. The experiments confirmed that Par-4 is involved in the apoptosis of islet *β* cells in type 2 diabetes.
3. Furthermore, we found that high glucose/palmitate intervention-induced endoplasmic reticulum stress can also promote the secretion of Par-4 and increased expression of Par-4 in islet *β* cells. Par-4 in the nucleus at the transcriptional level promoted NF-*κ*B expression and activity and promoted the apoptosis of islet *β* cells. At the same time, FAS and caspase-8 expression increased significantly. Overexpression of Par-4 could promote this process, and inhibition of Par-4 expression could inhibit this process. This indicated that the endoplasmic reticulum (ER) stress cell membrane approach could be a new pathway that is involved in the Par-4/NF-*κ*B induced islet *β* cell apoptosis. Previous research revealed that caspase-8 can shear and inactivate I*κ*B*α*, facilitating P65 translocation to the nucleus. Caspase-8 could be the key enzyme of the cell membrane apoptotic pathway, and this may be one of the mechanisms used by Par-4 and the cell membrane apoptotic pathway to induce the activation of NF-*κ*B [26].
4. We further found that the high glucose/palmitate intervention increased islet *β* cell nucleus Par-4 expression. The expression of caspase-9, the key enzyme of the mitochondrial apoptotic pathway, was increased significantly, and Bcl-2, the antiapoptotic factor, was significantly decreased. Cytochrome C release was increased significantly. Overexpression of Par-4 increased this process, and inhibition of Par-4 expression reduced this process, indicating that the mitochondrial pathway may also be a new pathway involved in the islet *β* cell apoptosis induced by Par-4/NF-*κ*B. Previous research revealed that fish oil inhibits the expression of caspase-9 and increases the expression of Bcl-2 to achieve the inhibition of NF-*κ*B. Another study also indicated that inhibition of caspase-3 resulted in decreased NF-*κ*B. All of the above indicated that Par-4 can activate the cell membrane and mitochondrial apoptosis pathways to induce the activation of NF-*κ*B [27, 28].

Based on the above results, we drew the following scientific conclusion: ER stress can activate the cell membrane apoptotic pathway and promote Par-4 secretion. Therefore, Par-4 enters the nucleus and causes the upregulation of NF-*κ*B activity to promote the apoptosis of islet *β* cells. Through the mitochondrial pathway, this also enables Par-4 to translocate into the nucleus and upregulate NF-*κ*B activity to promote apoptosis of the islet *β* cells.

**Figure 7:**
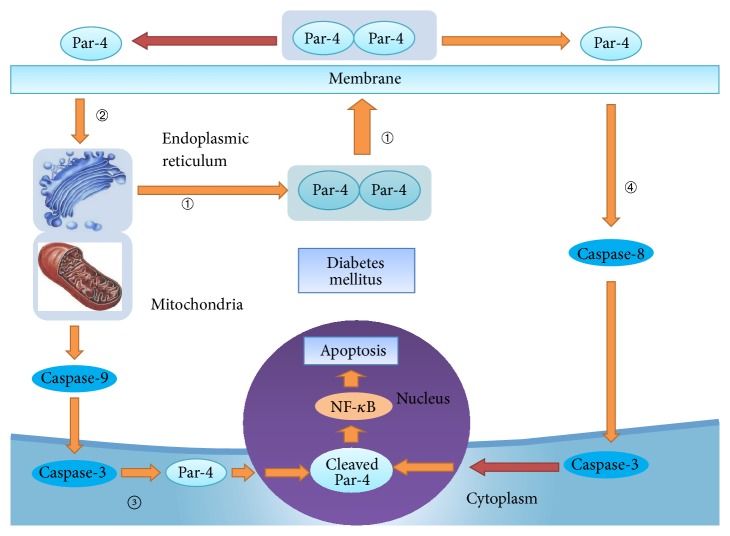
ER stress and mitochondrial dysfunction prompts Par-4/NF-*κ*B expression in the nucleus, increasing apoptosis of islet *β* cells after the high glucose/palmitate intervention. ① ER stress promotes the secretion of Par-4; ② the secretion of Par-4 increases ER stress; ③ mitochondrial dysfunction drives the translocation of Par-4 into the nucleus and induces cell apoptosis; ④ Par-4 translocates into the nucleus and induces cell apoptosis via the cell membrane pathway.

In summary, we conclude that Par-4/NF-*κ*B induces apoptosis in islet *β* cells via the ER stress cell membrane and mitochondrial pathways (See Figure 7). This gives us novel insight into the mechanism of apoptosis of islet *β* cells and identifies potential targets for the prevention and treatment of type 2 diabetes. This study provides new clues and ideas for the pathogenesis and the prevention of age-related diseases and also for biological engineering of cells and stem cells.

## 4. Material and Methods

### 4.1. The Construction of Adenovirus Vector

We used Oligo Designer 3.0 to design the 61-bp pawr primer template. After RT-PCR produced the pawr fragment, this pawr gene fragment was digested by EcoRI and BamHI and transfected to the linear pAdtrack-CMV vector. The recombinant pAdtrack-Par-4-CMV plasmid was obtained. The pAdTrack-Par-4-CMV plasmid and pAd-Easy-1 were cotransfected into competent* E. coli* to construct the recombinant adenovirus vector pAdTrack-Par-4 plasmid. All of the above were produced and the sequencing was performed by Shanghai China GenePharma Co., Ltd. Packing of the adenovirus using 293T cells and transduction of NIT-1 cells with the virus followed standard protocols.

### 4.2. Inhibition of Par-4

shRNA was designed using Dharmacon (http://dharmacon.gelifesciences.com/). After Blast evaluation, the sequence 5′GCAGATCGAGAAGAGGAAGCT 3′ was chosen, and the oligonucleotides were subcloned into the pAd-Easy-1 vector. All of the above were produced and the sequencing was performed by Shanghai China GenePharma Co., Ltd. Packing of the adenovirus using 293T cells and transduction of NIT-1 cells with the virus followed standard protocols.

### 4.3. Cell Culture and Reagents

The mouse insulinoma cell line NIT-1 was kindly provided by Dr. Chen Li Qing (TMMU, China). NIT-1 cells were grown in Dulbecco's modified Eagle's medium (DMEM, Hyclone, with a glucose concentration of 5 mmol/L DMEM), supplemented with 10% fetal bovine serum (FBS) (Hyclone) and 1% penicillin/streptomycin. Palmitic acids (Sigma) were dissolved at 100 mmol/L in DMEM medium containing 11% fatty acid-free bovine serum albumin (BSA) (Sigma) under an N_2_ atmosphere, shaken overnight at 37°C, sonicated for 15 min, and filtered under sterile conditions (stock solution). A control experiment was performed with fatty acids dissolved first in 10 mol/L NaOH and then diluted to 12 mmol/L in DMEM medium containing 12.5% fatty acid-free BSA (Sigma) under an N_2_ atmosphere and shaken overnight at 50°C. The calculated concentration of non-albumin-bound free fatty acid was 0.25 mmol/L palmitic acid for a final concentration. After incubation for 48 h, we used DMEM without FBS to induce cell cycle synchronization for 1 d in each group. The cells were divided into six groups: Group C (exposure of NIT-1 cells for 24 h to DMEM containing 12.5% BSA, with a glucose concentration of 5 mmol/L), Group C + Par-4 (exposure of NIT-1 cells for 24 h to DMEM containing 12.5% BSA with a glucose concentration of 5 mmol/L and then transfected with pAdTrack-Par-4 for 48 h), Group C − Par-4 (exposure of NIT-1 cells for 24 h to DMEM containing 12.5% BSA with a glucose concentration of 5 mmol/L and then treated with shRNA for Par-4 for 48 h), Group H (exposure of NIT-1 cells for 24 h to DMEM containing 12.5% BSA, with a glucose concentration of 25.5 mmol/L and 0.25 mmol/L palmitic acid), Group H + Par-4 (exposure of NIT-1 cells for 24 h to DMEM containing 12.5% BSA and 0.25 mmol/L palmitic acid and then transfected with pAdTrack-Par-4 for 48 h), and Group H − Par-4 (exposure of NIT-1 cells for 24 h to DMEM containing 12.5% BSA with a glucose concentration of 25.5 mmol/L and 0.25 mmol/L palmitic acid and then treated with by shRNA of Par-4 for 48 h), and, in 2.6, Group HP (exposure of NIT-1 cell for 24 h to DMEM containing 12.5% BSA and 0.25 mmol/L palmitic acid and then intervened by PDTC 50 *μ*mol/L for 48 h), Group H + Par-4 (overexpression of Par-4 and high glucose/palmitate intervention group), and Group HP + Par-4 (exposure of NIT-1 cell for 24 h to DMEM containing 12.5% BSA and 0.25 mmol/L palmitic acid and then intervened by PDTC 50 *μ*mol/L and transfected with pAdTrack-Par-4 for 48 h).

### 4.4. Immunohistochemistry

For antigen retrieval, the sections were microwaved in distilled water for 10 min followed by washing in phosphate-buffered saline (PBS) for 5 min. The deparaffinized sections were then incubated with primary antibodies as follows: Par-4 (dilution 1 : 200, Santa Cruz, USA) and NF-*κ*B (dilution 1 : 150, overnight at 4°C). The sections were washed with PBS and then incubated with horseradish peroxidase (HRP) labeled goat anti-rabbit antibody (dilution 1 : 100, DAKO, Glostrup, Denmark) for 30–60 min at room temperature. The sections were then washed three times in PBS and incubated at room temperature with 3,3′-diaminobenzidine (DAB) (DAKO, Glostrup, Denmark).

### 4.5. Western Blotting

After lysis buffer was added to cell, total protein was extracted, and the concentration was measured. Equal amounts of protein preparations were separated by sodium dodecyl sulfate-polyacrylamide gel electrophoresis (SDS-PAGE) for 30 min at 80 V; then, the separated proteins were transferred to nitrocellulose membranes (Boer Biotechnology Company) for 60 min at 120 V. The membranes were blocked with 5% nonfat milk (SIGMA, USA) in PBS with 0.05% Tween-20 (PBST, pH 7.6) for 2 h and then incubated with the following primary antibodies: Par-4 (1 : 400, Santa Cruz), GRP78 (1 : 300, Santa Cruz), and NF-*κ*B (P65) (1 : 500, Santa Cruz, USA) at 4°C overnight. The membranes were washed with TBST and then incubated with 1 : 5000 HRP-conjugated anti-rabbit IgG (Santa Cruz, USA) for 90 min on a tabletop incubator at 50 rpm and 37°C. The membranes were then washed again with TBST. The membranes were scanned with Typhoon (Pharmacia, USA) and quantitated using Quality One. We detected the protein expression level 3 times for each sample.

### 4.6. Apoptosis Detection

Cells were rinsed 3 times in PBS and then fixed in 4% paraformaldehyde. The samples were then incubated with TUNEL solution for 90 min at 37°C. Adding a sodium citrate solution terminated the reaction. The samples were then incubated with anti-HRP antibody for 15 min, washed with PBS, and stained with DAB (Figure 6(b)) or DAB and hematoxylin (Figure 2(b)). The samples were observed and photographed at 400x on a light microscope and the apoptosis rate was calculated as previously described [29].

### 4.7. Measurement of Insulin after Glucose Stimulation

Cells were plated in 48-well plates at a density of 1 × 10^5^ cells/well. After 16 h, the medium was removed; the cells were washed once and then incubated for 1 h in glucose-free Krebs-Ringer bicarbonate (KRB) buffer (115 mM NaCl, 4.7 mM KCl, 1.2 mM MgSO_4_·7H_2_O, 1.2 mM KH_2_PO_4_, 20 mM NaHCO_3_, 16 mM HEPES, 2.56 mM CaCl_2_, and 0.2% BSA). The cells were then treated for 1 h in KRB buffer with low high (25 mM) glucose. The medium was collected and stored at −20°C for ELISA (using a kit, EZRMI-13K, Millipore) to determine insulin secretion. For determining the insulin content, the cells were lysed with 0.1% Triton X-100. The insulin content and secretion were normalized to the total protein content using the bicinchoninic acid method. Changes in insulin secretion and content were then calculated with reference to the results obtained from the control group.

### 4.8. Measurement of Cytochrome C

Cytochrome C was detected with a cytochrome C (MBL). Cells were lysed in the cold preparation buffer included in the ELISA kit. Cell homogenates were centrifuged (10,000 ×g for 1 h at 4°C) and collected. Protein concentration was assayed by the bicinchoninic acid assay (BCA). The samples were then treated with a conjugated reagent, transferred to a cytochrome C antibody-coated microwell plate, and incubated for 1 h at room temperature. Next, the plate was washed and treated with a substrate reagent and incubated for 0.5 h, followed by the addition of Stop Solution. The absorbance was read at 450 nm with an automatic microplate reader (Spectra Max, M5, Molecular Devices, USA). Serial dilutions of a cytochrome C calibrator were assayed along with the samples to establish a standard curve, which was used to calculate the concentration of cytochrome C.

### 4.9. Measurement of Secretory Par-4

Secretory Par-4 was detected with a Par-4 ELISA kit (MBL). Cell supernatant was collected. Protein concentration was assayed by the bicinchoninic acid assay (BCA). The samples were then treated with a conjugated reagent, transferred to Par-4 antibody-coated microwell plate, and incubated for 1 h at room temperature. Next, the plate was washed and treated with a substrate reagent and incubated for 0.5 h, followed by the addition of Stop Solution. The absorbance was read at 450 nm with an automatic microplate reader (Spectra Max, M5, Molecular Devices, USA). Serial dilutions of a Par-4 calibrator were assayed along with the samples to establish a standard curve, which was used to calculate the concentration of Par-4.

### 4.10. Electrophoretic Mobility Shift Assay (EMSA)

Nuclear extracts were prepared using NE-PERTM Nuclear and Cytoplasmic Extraction Reagents (Pierce). The following oligonucleotide contained a consensus NF-*κ*B binding site: 5′-AGTTGAGGGGACTTTCCCAGGC-3′. It was end-labeled with the Biotin 3′-End DNA Labeling Kit (Pierce) and employed using a LightShift Chemiluminescent EMSA Kit (Pierce). Binding reactions were performed as follows: nuclear extracts (10 *μ*g of protein) and 1x binding buffer with 2.5% glycerol, 5 mmol/L MgCl_2_, 50 ng/*μ*L poly(dI-dC), 0.05% NP-40, and 20 fmol biotin 3′-end-labeled double-stranded oligonucleotides were incubated on ice for 20 min in a volume of 20 *μ*L. DNA-protein complexes were resolved on nondenaturing 6% polyacrylamide gels at 100 V for 2 h. After gel electrophoresis, the DNA-protein complexes were transferred to a positively charged nylon membrane (Pharmacia, USA).

### 4.11. Statistical Analysis

The data are shown as the mean ± standard deviation (*X* ± *S*) and were analyzed using SPSS 19.0. The nonparametric rank sum test and analysis of variance (ANOVA) were used and *P* < 0.05 indicated a significant difference.

## Figures and Tables

**Table 1:** The insulin secretion of each group.

	C	C + Par-4	C − Par-4	H	H + Par-4	H − Par-4
Insulin (mIU/L)	6.67 ± 0.24^**∗**&#^	6.43 ± 0.30	6.65 ± 0.36	3.60 ± 0.20^&#^	2.29 ± 0.08^**∗**&^	4.48 ± 0.26^**∗**#^

^*∗*^Compared with H group; *P* < 0.05.

^#^Compared with H − Par-4 group; *P* < 0.05.

^&^Compared with H + Par-4 group; *P* < 0.05.

**Table 2:** The insulin secretion, Par-4 secretion, apoptosis rate, and protein expression of GRP78 and Par-4 of each group.

	C	C + Par-4	C − Par-4	H	H + Par-4	H − Par-4
Insulin (mIU/L)	6.67 ± 0.24^*∗*&#^	6.43 ± 0.30	6.65 ± 0.36	3.60 ± 0.20^&#^	2.29 ± 0.08^*∗*&^	4.48 ± 0.26^*∗*#^
Par-4 (mg/L)	70.05 ± 14.18^*∗*&#^	71.78 ± 19.38	67.60 ± 16.32	388.18 ± 18.43^&#^	766.22 ± 16.15^*∗*&^	164.58 ± 16.63^*∗*#^
Apoptosis rate (%)	2.88 ± 1.25^*∗*&#^	3.13 ± 1.55	2.13 ± 1.13	45.38 ± 7.91^&#^	62.75 ± 10.55^*∗*&^	31.63 ± 6.00^*∗*#^
GRP78	0.14 ± 0.01^*∗*&#^	0.14 ± 0.04	0.15 ± 0.03	0.89 ± 0.05^&#^	1.18 ± 0.13^*∗*&^	0.60 ± 0.10^*∗*#^
Par-4	0.81 ± 0.03^*∗*&#^	1.11 ± 0.03	0.63 ± 0.03	1.67 ± 0.10^&#^	2.22 ± 0.08^*∗*&^	0.91 ± 0.04^*∗*#^

^*∗*^Compared with H group; *P* < 0.05.

^#^Compared with H − Par-4 group; *P* < 0.05.

^&^Compared with H + Par-4 group; *P* < 0.05.

**Table 3:** The nuclear protein expression of Par-4 and NF-*κ*B of each group.

	C	C + Par-4	C − Par-4	H	H + Par-4	H − Par-4
Par-4	0.67 ± 0.01^*∗*&#^	0.82 ± 0.11	0.47 ± 0.11	1.11 ± 0.05^&#^	1.37 ± 0.01^*∗*&^	0.95 ± 0.01^*∗*#^
NF-*κ*B	1.81 ± 0.09^*∗*&#^	1.76 ± 0.09	1.78 ± 0.08	2.46 ± 0.15^&#^	2.96 ± 0.21^*∗*&^	2.13 ± 0.23^*∗*#^

^*∗*^Compared with H group; *P* < 0.05.

^#^Compared with H − Par-4 group; *P* < 0.05.

^&^Compared with H + Par-4 group; *P* < 0.05.

**Table 4:** The protein expression of FAS and caspase-8 of each group.

	C	C + Par-4	C − Par-4	H	H + Par-4	H − Par-4
FAS	0.35 ± 0.03^*∗*&#^	0.33 ± 0.07	0.35 ± 0.05	0.58 ± 0.04^&#^	0.69 ± 0.03^*∗*&^	0.49 ± 0.06^*∗*#^
Caspase-8	0.41 ± 0.04^*∗*&#^	0.42 ± 0.03	0.42 ± 0.04	0.60 ± 0.04^&#^	0.67 ± 0.04^*∗*&^	0.52 ± 0.04^*∗*#^

^*∗*^Compared with H group; *P* < 0.05.

^#^Compared with H − Par-4 group; *P* < 0.05.

^&^Compared with H + Par-4 group; *P* < 0.05.

**Table 5:** The cytochrome C secretion and protein expression of Bcl-2 and caspase-9 of each group.

	C	C + Par-4	C − Par-4	H	H + Par-4	H − Par-4
Cytochrome C (ng/mL)	16.29 ± 4.44^*∗*&#^	17.19 ± 4.19	17.93 ± 4.27	67.76 ± 4.46^&#^	113.68 ± 6.85^*∗*&^	37.97 ± 7.89^*∗*#^
Bcl-2	0.63 ± 0.03^*∗*&#^	0.63 ± 0.03	0.64 ± 0.04	0.42 ± 0.01^&#^	0.32 ± 0.03^*∗*&^	0.54 ± 0.09^*∗*#^
Caspase-9	0.44 ± 0.04^*∗*&#^	0.41 ± 0.03	0.43 ± 0.04	0.67 ± 0.04^&#^	0.80 ± 0.07^*∗*&^	0.59 ± 0.02^*∗*#^

^*∗*^Compared with H group; *P* < 0.05.

^#^Compared with H − Par-4 group; *P* < 0.05.

^&^Compared with H + Par-4 group; *P* < 0.05.

**Table 6:** The apoptosis rate and nuclear protein expression of Par-4 and NF-*κ*B of each group.

	C	H	H + Par-4	HP	HP + Par-4
Apoptosis rate (%)	2.88 ± 1.25^*∗*&#^	45.38 ± 7.91^&#^	62.75 ± 10.55^*∗*#^	36.38 ± 5.60^*∗*&^	37.88 ± 6.45^*∗*&^
Par-4	1.06 ± 0.17^*∗*&#^	2.03 ± 0.05^&#^	2.49 ± 0.04^*∗*#^	2.10 ± 0.12^*∗*&^	2.59 ± 0.19^*∗*&^
NF-*κ*B	0.57 ± 0.03^*∗*&#^	1.12 ± 0.03^&#^	1.53 ± 0.08^*∗*#^	0.64 ± 0.05^*∗*&^	0.65 ± 0.14^*∗*&^

^*∗*^Compared with H group; *P* < 0.05.

^#^Compared with HP + Par-4 group; *P* < 0.05.

^&^Compared with H + Par-4 group; *P* < 0.05.
